# Examining the Role of a Comprehensive eConsult System to Enhance Access to Nephrology Care

**DOI:** 10.1016/j.xkme.2026.101287

**Published:** 2026-02-10

**Authors:** Elizabeth Cheng, Kevin X. Shi, Delphine S. Tuot

**Affiliations:** 1 University of California, Berkeley, Berkeley, CA; 2 Division of Nephrology, Department of Medicine, University of California San Francisco, San Francisco, CA; 3 Center for Innovation in Access and Quality at Zuckerberg San Francisco General Hospital, University of California San Francisco, San Francisco, CA

**Keywords:** Access to care, electronic consultation, kidney disease, nephrology, safety-net hospitals

## Abstract

**Rationale & Objective:**

A stagnant US nephrology workforce and rising chronic kidney disease (CKD) prevalence underscore the need for improved nephrology expertise accessibility. We examined nephrology consultation patterns in one health care setting with a comprehensive eConsult program in which all specialty care requests are initiated via eConsult. Results may inform the development of interventions to better support referring clinicians.

**Study Design:**

A cross-sectional study examining a random sample of ambulatory nephrology eConsults (12.8%, n = 450/3,514) submitted January 1, 2020 to December 31, 2023.

**Setting & Population:**

A publicly funded institution providing health care services to residents of San Francisco, California.

**Predictor:**

Characteristics of eConsults, including clinical question type (ie, management, diagnosis, and medication safety), inquiry topics (ie, CKD, proteinuria, and hypertension) and referring provider (ie, MD/DO or advanced practice provider).

**Outcomes:**

Disposition of eConsults (ie, not scheduled for nephrology visit, scheduled immediately after eConsult review, or scheduled after asynchronous dialogue between referring provider and nephrologist reviewer).

**Analytical Approach:**

Univariate and multivariable logistic regression models (adjusted for patient age, sex, and kidney function) assessed associations between eConsult characteristics and disposition, defined as a binary outcome (scheduled vs not scheduled).

**Results:**

Nearly half of eConsults (47%, n = 211) were resolved without nephrology appointments. Of the eConsults, 31% (n = 139) were scheduled immediately and 22% (n = 100) after consultative dialogue. CKD (53%, n = 239), proteinuria (26%, n = 115), and hypertension (17%, n = 75) were the most queried topics. Compared with diagnostic submissions, management-related eConsults were more likely to be scheduled (adjusted OR, 2.07; 95% CI, 1.25-3.45), whereas medication-related questions were less likely to be scheduled (adjusted OR, 0.14; 95% CI, 0.03-0.64).

**Limitations:**

Generalizability of findings is limited by the single-center design.

**Conclusions:**

By resolving nearly half of consult requests asynchronously, eConsult can enhance nephrology expertise accessibility for populations experiencing high CKD burden and limited availability of synchronous nephrology care.

## Plain-Language Summary

Access to nephrology care is limited in the United States, particularly in safety-net health systems that serve uninsured and underinsured populations. To inform the development of interventions to better support referring clinicians and improve accessibility of nephrology care, we examined nephrology consultation patterns in one health care delivery system that uses a comprehensive eConsult platform in which all specialty consultation requests are initiated with an eConsult. We found that nearly half of nephrology eConsults were resolved without the scheduling of a nephrology appointment, including consultation requests about CKD diagnostic approaches and medication safety. With triaging and educational functionalities, a comprehensive eConsult program may enhance timely access to nephrology care in low-income populations.

Electronic consultation (eConsult) programs allow referring providers and specialists to asynchronously exchange clinical information regarding patient-specific concerns.^1^ Through eConsults, specialists have the opportunity to address inquiries and advance individualized patient care plans without a scheduled patient visit, potentially improving access for high complexity patients who benefit most from synchronous in-person or telehealth specialty care visits.^2^ eConsult programs also expand the scope of practice for primary care providers (PCPs) and increase knowledge through collaboration and interaction with specialists in the eConsult workflow.^3^

Chronic kidney disease (CKD) affects an estimated 850 million individuals worldwide,^4^ and its progression may lead to kidney failure, necessitating dialysis or kidney transplantation. Given the similarities between CKD care and that of diabetes and hypertension, 2 conditions routinely addressed by PCPs, many cases of early-stage CKD can be managed in primary care settings.^5^ However, PCPs have low self-efficacy or confidence in the management of CKD.^6^ With the current stagnant state of the US nephrology workforce^7^ and the increasing prevalence of CKD, eConsults have the potential to play an outsized role in ensuring appropriate care for patients with early CKD.^8^ eConsult programs may be of particular importance for safety-net settings that serve racially, ethnically, and linguistically diverse low-income patients who have a high burden of CKD and experience even more limited access to timely nephrology services.^9^

A few nephrology eConsult programs in the United States and Canada that exist in parallel to traditional referral systems have been shown to enhance access to timely nephrology support and reduce unnecessary outpatient nephrology visits.^10^, ^11^, ^12^ To our knowledge, very few studies have examined the role of a comprehensive eConsult program in which all ambulatory referrals to nephrology care are initially submitted as an eConsult. This type of eConsult system can provide a complete view of all requests within the organization for nephrology expertise, potentially informing the development of interventions to better support PCPs and improve accessibility of nephrology care for high-risk patients, such as those likely to progress to kidney failure, requiring specialized management, or facing diagnostic challenges. We sought to bridge this gap by examining the types of consult questions submitted to the nephrology service and their association with the need for a synchronous nephrology visit in one health care delivery system with a comprehensive eConsult program. We hypothesized that at least one-third of eConsult requests could be managed without a synchronous patient visit and that those eConsults initiated to clarify or establish a nephrology diagnosis would be more likely to result in the scheduling of a nephrology visit than those focused on management of an existing diagnosis, due to the potential need for clinical evaluation in the diagnostic process.

## Methods

### Design

We performed a cross-sectional analysis on a randomly selected deidentified sample (12.8%, n = 450/3,514) of all ambulatory consultation requests submitted to the nephrology service between January 1, 2020 and December 31, 2023. This sample size was selected to balance the feasibility of manual review of each eConsult with the objective of capturing a diverse and representative subset of consultation patterns within the study setting. This study was considered exempt by the University of California, San Francisco institutional review board.

### Setting and Patient Population

The Zuckerberg San Francisco General Hospital and Trauma Center is a facility within the San Francisco Health Network, which is an integrated public health care delivery system that provides comprehensive health care services to uninsured and underinsured San Francisco residents. In 2022-2023, 155,000 in-person and 23,000 telehealth specialty office visits took place at Zuckerberg San Francisco General Hospital and Trauma Center, largely supported by public health insurance: 58% Medi-Cal, 23% Medicare, 8% uninsured, and 11% other. The patient population at Zuckerberg San Francisco General Hospital and Trauma Center is racially and ethnically diverse, with 42% Latino/a or Hispanic, 20% Asian, 16% White, 13% Black or African American, and 9% other.

### eConsult Program

San Francisco Health Network eConsult is an integrated referral and consultation platform that was started in 2005 to improve the efficiency of specialty care delivery.^13^ In this system, all requests for specialty care are submitted exclusively via the eConsult platform. When appropriate, eConsults are assigned the billing code 99451 for internal documentation and tracking but are not submitted for reimbursement in this safety-net setting. The program maintains a system-wide performance goal of responding to 90% of submitted eConsults within 5 days; in routine practice, 91% of submissions receive a response within 3 business days.^14^

When generating an eConsult to a specified specialty service, a referring provider can input brief clinical questions or indications contextualized with pertinent patient information, including clinical history, diagnostic data, symptoms, and physical exam findings, in text entry fields. Following the eConsult submission, a specialist reviewer can provide written clinical guidance for co-management of patient care, request supplementary patient information and recommend diagnostic testing for further assessment, or initiate the scheduling of a specialty appointment. For each approach, the referring provider and specialist reviewer can exchange asynchronous dialogue until the referring provider closes the eConsult.

eConsults submitted to the nephrology service are reviewed by a single nephrologist, with coverage provided by 1 additional nephrologist as needed. Responses to eConsults are composed at the discretion of the reviewing nephrologist based on the clinical context of each submission and are not guided by a standardized template.

### Data Analysis

For each case that is submitted, the eConsult system automatically records the patient’s demographics, including age, legal sex, preferred language, and race and ethnicity. Asynchronous dialogue exchanged between the referring provider and specialist reviewer is also documented. All data, including the clinical inquiries and associated patient information from the 3,514 eConsults submitted to the nephrology service between January 1, 2020 and December 31, 2023 were obtained. Of the 3,514 eConsults retrieved from this 4-year period, 450 were randomly selected to be examined for this study using a computer-generated random number algorithm in R Version 4.4.0 (R Foundation for Statistical Computing).

Following deidentification, data from the randomly selected sample of eConsults were transferred to REDCap LTS Version 14.0.36 (Research Electronic Data Capture, Vanderbilt University) for analysis. Two independent reviewers (E.C., D.S.T.) manually classified the submissions by clinical question type in accordance with a validated taxonomy.^15^ The categories were mutually exclusive and included diagnosis, management, and medication-related inquiries. eConsults were also classified according to the following variables: (1) general topics of inquiry, with nonexclusive categories of CKD, proteinuria (including albuminuria), electrolyte or acid-base imbalance, acute kidney injury, hypertension, glomerulonephritis, urinary obstruction or stones, and renal masses or cysts; (2) disposition of the eConsult, with mutually exclusive categories of not scheduled for an in-person or telehealth nephrology visit prior to closure of the eConsult, scheduled immediately after the eConsult review, or scheduled after asynchronous dialogue between the referring provider and nephrologist reviewer; and (3) type of referring provider, with mutually exclusive categories of MD/DO, advanced practice provider, referral coordinator, or PharmD. Each variable contained an ‘Other,’ or ‘Unknown’ in the case of the type of referring provider, category in addition to the predefined categories to accommodate deviations. Any variance in reviewer assessments were addressed collaboratively.

Descriptive statistics were generated using Stata 14 (StataCorp LLC) to evaluate the usage patterns of the eConsult program. Univariate associations between eConsult characteristics (clinical question type, referring provider, and topics of inquiry) and disposition were assessed using the χ^2^ test. The independent association between clinical question type (independent variable) and disposition (binary variable: scheduled vs not scheduled for a nephrology visit prior to closure of the eConsult) was evaluated using multivariable logistic regression, adjusted for patient age at referral (continuous variable), sex, and serum creatinine (continuous variable), which were determined a priori. All tests were conducted with a 2-tailed approach, and *P* values <0.05 were considered statistically significant.

## Results

### Patient Demographics

Within the sample of 450 patients for whom eConsults were submitted to nephrology, 14% (n = 61) were aged ≤40 years, 54% (n = 242) were >60, and 58% (n = 259) were male. One-quarter of patients (25%, n = 114) identified as Black or African American, 22% (n = 99) as Asian, 16% (n = 72) as White, 3% (n = 13) as Native Hawaiian or other Pacific Islander, and 33% (n = 147) as other or multiracial. Nearly one-third (28%; n = 127) of patients were of Latino/a or Hispanic origin. English was the preferred language for nearly two-thirds (64%, n = 287) of patients, followed by Spanish (20%, n = 90) and Chinese (9%, n = 40).

A recent serum creatinine test result was available for the majority (93%, n = 417) of eConsult patients. The mean ± standard deviation serum creatinine level was 1.83 ± 1.54 mg/dL and the median was 1.4 mg/dL. One-half (50%, n = 227) of patients had results from a recent urinary albumin-creatinine ratio test. The mean level was 1,206.96 ± 2,182.82 mg/g and the median was 272.3 mg/g.

Demographic and clinical characteristics of patients for whom eConsults were submitted are listed in Table 1. They were comparable to those describing patients whose eConsults were not included in the analytical sample, with the exception of sex. eConsults from male patients were slightly under-represented in the analytic sample (57.6% vs 60.3%, *P* = 0.02; Table S1).

**Table 1:** Demographics and Clinical Characteristics of Patients for Whom eConsults Were Submitted and Analyzed (N = 450)

Variable	n (%) or Mean ± SD
Age, y	
≤40	61 (13.6%)
41-60	147 (32.7%)
>60	242 (53.8%)
Legal sex	
Male	259 (57.6%)
Female	190 (42.2%)
Nonbinary	1 (0.22%)
Race	
Black/African American	114 (25.3%)
Asian	99 (22.0%)
White	72 (16.0%)
Native Hawaiian/other Pacific Islander	13 (2.9%)
American Indian/Alaska Native	2 (0.4%)
Other/multiracial	147 (32.7%)
Decline to state	3 (0.7%)
Ethnicity	
Hispanic/Latino/Spanish	127 (28.2%)
Not Hispanic/Latino/Spanish	322 (71.6%)
Decline to state	1 (0.2%)
Preferred language	
English	287 (63.8%)
Spanish	90 (20.0%)
Chinese	40 (8.9%)
Other	31 (6.9%)
Decline to state	2 (0.4%)
Clinical characteristics	
Serum creatinine, mg/dL (n = 417)	1.83 ± 1.54
UACR, mg/g (n = 227)	1,206.96 ± 2,182.82

Abbreviations: SD, standard deviation; UACR, urinary albumin-creatinine ratio.

### eConsult Characteristics

Three-quarters (76%, n = 340) of eConsult inquiries were concerned with overall management, 19% (n = 86) with diagnosis, and 5% (n = 22) with medication safety/dosing; the remaining 0.4% (n = 2) were classified as ‘Other.’ Of the eConsults pertaining to diagnosis, 80% (n = 69) inquired about types of tests to order, and 20% (n = 17) inquired about interpreting test results. The majority of eConsults were pertinent to the topic of CKD (53%, n = 239). Proteinuria (26%, n = 115) and hypertension (17%, n = 75) were the second and third most common topics queried in the eConsults.

For 47% (n = 211) of eConsults, no specialty visit was scheduled with a nephrologist before closure of the eConsult. Fewer than one-third (31%, n = 139) were scheduled immediately upon specialty review, and 22% (n = 100) were scheduled after asynchronous written consultative exchange between the referring provider and nephrologist reviewer. The most common referring provider types were MD/DOs (60%, n = 272) and advanced practice providers (22%, n = 101). Of the referring provider types, 12% (n = 54) were unknown. Table 2 depicts the full characteristic details of the eConsults reviewed for this study.

**Table 2:** Characteristics of the Randomly Selected Sample of eConsults Analyzed (N = 450)

Characteristic	n (%)
Clinical question type	
Management	340 (75.6%)
Diagnosis	86 (19.1%)
Inquiries on types of tests to order	69 (15.3%)
Inquiries on interpreting test results	17 (3.8%)
Medication inquiry	22 (4.9%)
Other	2 (0.4%)
Topics of inquiry^a^	
Chronic kidney disease	239 (53.1%)
Proteinuria	115 (25.6%)
Hypertension	75 (16.7%)
Acute kidney injury	58 (12.9%)
Electrolyte/acid-base imbalance	45 (10.0%)
Urinary obstruction/stones	18 (4.0%)
Glomerulonephritis	15 (3.3%)
Renal mass/cyst	6 (1.3%)
Other	83 (18.4%)
Disposition	
Not scheduled	211 (46.9%)
Initially scheduled	139 (30.9%)
Scheduled after provider communication	100 (22.2%)
Referring provider	
MD/DO	272 (60.4%)
APP	101 (22.4%)
PharmD	6 (1.3%)
Referral coordinator	17 (3.8%)
Unknown	54 (12.0%)

Abbreviation: APP, advanced practice provider.

a Percentages sum to >100% as eConsults could be coded into multiple topics of inquiry.

### Disposition Across eConsult Characteristics

In univariate analysis, differences were observed in the disposition of the eConsults by the type of clinical question (Table 3). eConsults with inquiries about management were most likely to be scheduled for a nephrology appointment, either initially or after provider communication (59%, n = 202/340), while the majority (91%, n = 20/22) of medication inquiries were not scheduled. There were also differences found in eConsult disposition among types of referring providers. eConsults submitted by MD/DOs and referral coordinators more commonly resulted in patients being scheduled either immediately upon review or after asynchronous dialogue (53%, n = 143/272 and 53%, n = 9/17, respectively), when compared with advanced practice providers (48%, n = 48/101) or PharmDs (17%, n = 1/6). Among the clinical topics of inquiry, eConsults concerning glomerulonephritis were the most likely to be scheduled for a nephrology appointment either initially or after communication between providers (87%, n = 13/15), followed by CKD (62%, n = 147/239) and proteinuria (59%, n = 68/115). The majority of submissions inquiring about urinary obstruction or stones (67%, n = 12/18) and renal masses or cysts (67%, n = 4/6) were resolved asynchronously.

**Table 3:** eConsult Disposition by Clinical Question Type, Referring Provider Type, and Topics of Inquiry

Characteristic	Disposition					
	Not Scheduled (N = 211)		Initially Scheduled (N = 139)		Scheduled After Provider Communication (N = 100)	
	n	%	n	%	n	%
Clinical question type						
Management	138	40.6%	124	36.5%	78	22.9%
Diagnosis	51	59.3%	15	17.4%	20	23.3%
Medication inquiry	20	90.9%	0	0.0%	2	9.1%
Other	2	100.0%	0	0.0%	0	0.0%
Referring provider						
MD/DO	129	47.4%	79	29.0%	64	23.5%
APP	53	52.5%	21	20.8%	27	26.7%
PharmD	5	83.3%	0	0.0%	1	16.7%
Referral coordinator	8	47.1%	4	23.5%	5	29.4%
Unknown	16	29.6%	35	64.8%	3	5.6%
Topics of inquiry^a^						
Chronic kidney disease	92	38.5%	88	36.8%	59	24.7%
Proteinuria	47	40.9%	36	31.3%	32	27.8%
Hypertension	35	46.7%	21	28.0%	19	25.3%
Acute kidney injury	24	41.4%	19	32.8%	15	25.9%
Electrolyte/acid-base imbalance	27	60.0%	7	15.6%	11	24.4%
Urinary obstruction/stones	12	66.7%	3	16.7%	3	16.7%
Glomerulonephritis	2	13.3%	10	66.7%	3	20.0%
Renal mass/cyst	4	66.7%	0	0.0%	2	33.3%
Other	42	50.6%	21	25.3%	20	24.1%

Abbreviation: APP, advanced practice provider.

a Number of eConsults sum to greater than the total as eConsults could be coded into multiple topics of inquiry.

Multivariable analysis suggested that eConsults pertaining to management had increased odds of being scheduled (either initially or after consultative communication) than eConsults inquiring about diagnosis (adjusted OR, 2.07; 95% CI, 1.25-3.45), independent of patient age, sex, and serum creatinine level (Table S2). On the contrary, eConsults submitted for medication inquiries had higher odds of not being scheduled for an appointment than those about diagnosis (adjusted OR, 0.14; 95% CI, 0.03-0.64).

## Discussion

This study identified 3 key findings regarding the use of eConsults for nephrology care in a safety-net health care system with a comprehensive eConsult program: (1) nearly half of eConsults submitted to nephrology were resolved without requiring the scheduling of an in-person or telehealth nephrology appointment; (2) eConsults that inquired about management strategies to optimize kidney health and diagnosis were more likely to be scheduled for a nephrology appointment, whereas those about medication safety and appropriateness were more likely to be addressed asynchronously; and (3) among the topics broached in eConsult submissions, CKD was the most frequent, followed by proteinuria and hypertension.

Our finding that nearly half of eConsults were resolved asynchronously rather than through the scheduling of a specialty appointment is consistent with prior studies,^10^^,^^11^ which found that nephrology eConsult programs that exist in parallel with traditional referral systems result in a substantial reduction in the number of specialty visits scheduled. This suggests that all types of eConsult programs may result in shorter wait times for nephrology visits compared with scheduling an appointment through a traditional referral mechanism. We found that a large proportion of eConsult submissions that were scheduled for a nephrology visit were concerned with disease management. This was initially surprising to us, as management of lower complexity nephrology topics such as early CKD is feasible in primary care settings, whether by PCPs independently or through asynchronous co-management with a nephrologist, given the wide availability of structured data to guide clinical decision making, such as glomerular filtration rate, proteinuria, and blood pressure. Our comprehensive eConsult program is over a decade old; although speculative, it is possible that referring providers have gained nephrology knowledge over the years about lower-complexity CKD management topics through the eConsult program^3^ and that recent management inquiries pertain to higher-complexity cases that require comprehensive evaluation and synchronous specialized nephrology expertise. Study participant median serum creatinine of 1.8 mg/dL lends credibility to this hypothesis, as this usually represents moderate CKD (vs early CKD). eConsults about diagnosis were also frequently scheduled for an appointment with a nephrologist. This is aligned with the emphasis in recent Kidney Disease: Improving Global Outcomes (KDIGO) guidelines^16^ to identify the etiology of CKD, which often benefits from a physical exam and a detailed patient history. Unsurprisingly, the majority of eConsults pertaining to medication appropriateness were resolved asynchronously and did not result in a scheduled nephrology appointment.

The 2 most queried topics in eConsult submissions included CKD and proteinuria/albuminuria. This finding is consistent with recent qualitative data from semistructured interviews of PCPs who identified a need for more education about new classes of medications to slow CKD decline and minimize proteinuria, including sodium/glucose cotransporter 2 inhibitors, glucagon-like peptide 1 receptor agonists, and nonsteroidal mineralocorticoid receptor antagonists.^17^ PCPs hold critical roles in the management of CKD, treatment of associated comorbid conditions, and prevention of disease progression and complications.^18^ The introduction of newer pharmacologic interventions can present challenges in providing each patient with CKD with the most effective and evidence-based care. Ensuring that PCPs are confident in their ability to effectively incorporate these new medications into management plans may enhance CKD care in primary care settings and improve patient outcomes. Local continuing medical education programs that focus on commonly queried clinical topics (in our system: CKD, proteinuria, and hypertension) and consultative responses via the eConsult program could reinforce PCP knowledge and aid in bridging this educational gap. Proactive electronic consultation systems could also serve as educational endeavors.^17^^,^^19^

These findings build upon those of prior studies about electronic consultation by highlighting the potential role of an eConsult program for nephrology care delivery in settings with low nephrologist availability for synchronous patient care. Low-income patients who receive care in safety-net clinics are either uninsured or have public insurance; they experience more restricted access to timely nephrology services than individuals with private insurance and have been found to have disproportionately high rates of CKD and a greater burden of associated morbidity and mortality.^9^ These disparities underscore the critical need for interventions that enhance the delivery of nephrology expertise to safety-net populations with early CKD who may not require a synchronous nephrology visit, as well as those with more advanced CKD who are at highest risk of kidney-related complications. Our findings suggest that a comprehensive eConsult program with triage and co-management functionalities may be one example of such an intervention.

Limitations of our study include its completion within a single health care system, which restricts the generalizability of our findings to other health systems. In our setting, all nephrology care requests are initiated via eConsult, which allows for asynchronous specialist expertise and in-person appointments when necessary. Referring providers are aware that not all eConsults will result in scheduled appointments, potentially influencing the types of consultative questions they submit to the nephrology service. However, prior studies have demonstrated that transitioning from a traditional referral model to an eConsult-based system does not significantly alter the total number of consultation requests submitted.^20^ This suggests the broader utilization patterns of eConsult and traditional referral processes may be consistent. In addition, without data on how the eConsult program affects the delivery of nephrology care, such as its impact on wait times for appointments, patient safety, or quality of care, we are unable to draw conclusions about the long-term effects of the platform. Our study also did not specifically address provider experience with the eConsult platform, including nephrologist and referring provider time spent replying to asynchronous messages or satisfaction with the services, although prior studies of our global eConsult system as well as others across the United States have demonstrated high provider satisfaction with eConsult platforms.^14^ Additionally, while the random sample of eConsults analyzed in our study is the largest to date on this topic, it accounted for only 12.8% of eConsults submitted to the nephrology service during the study period. This may not be a representative sample and could introduce selection bias.^21^ In future studies, use of high-throughput textual analysis methods, such as artificial intelligence and natural language processing technologies, may allow for larger sample analysis. These technologies may also enable higher resolution classification than traditional taxonomical strategies, setting the grounds for more targeted educational opportunities.

## Conclusions

The comprehensive eConsult program examined in our study potentially halved the number of nephrology appointments requested by allowing referring providers to receive timely asynchronous nephrology expertise. eConsults concerning management were the most likely to be scheduled for a nephrology visit; those concerning medication safety and appropriateness were the least likely to be scheduled. Further research to evaluate the lasting outcomes of comprehensive eConsult programs on quality of specialty care delivery is needed to inform improvements to specialty care access, patient services, and care coordination, particularly for low-income populations with the least access to nephrology care.
