# Supplementary material for: Examining the Role of a Comprehensive eConsult System to Enhance Access to Nephrology Care
Source: Kidney Med. 2026 Feb 10;8(4):101287. doi: 10.1016/j.xkme.2026.101287 (PMC12992938; doi:10.1016/j.xkme.2026.101287)
Supplement: Supplementary File (PDF) — Table S1-S2. [file mmc1.pdf]

Tables S1. Demographics and clinical characteristics of patients for whom eConsults were not included in the analytical sample compared to those included in the analytic sample.

Variable	*N* = 3064		*p*-Value (Analytical vs. Non-Analytical Sample)
**Age, *n* (%)**			0.88
≤40 years	390	(12.7)	
41-60 years	1019	(33.3)	
>60 years	1655	(54.0)	
**Legal Sex, *n* (%)**			0.02
Male	1847	(60.3)	
Female	1217	(39.7)	
Nonbinary	0	(0.0)	
**Race, *n* (%)**			0.25
Black/African American	666	(21.8)	
Asian	816	(26.7)	
White	572	(18.7)	
Native Hawaiian/other Pacific Islander	80	(2.6)	
American Indian/Alaska Native	13	(0.4)	
Other/multiracial	895	(29.3)	
Decline to state	17	(0.6)	
**Ethnicity, *n* (%)**			0.27
Hispanic/Latino/Spanish	778	(25.4)	
Not Hispanic/Latino/Spanish	2275	(74.4)	
Decline to state	7	(0.2)	
**Preferred Language**			0.13
English	1886	(61.6)	
Spanish	540	(17.6)	
Chinese	312	(10.2)	
Other	326	(10.6)	
Decline to state	0	(0.0)	
**Clinical Characteristics**, mean (SD)**			
sCr (mg/dL)	1.98	(1.74)	0.10
uACR (mg/g)	1289.45	(2440.46)	0.63

sCr: serum creatinine; SD: standard deviation; uACR: urine albumin-creatinine ratio. **sCr *n* = 2816, uACR *n* = 1391

Table S2. Adjusted odds ratios and 95% confidence intervals for eConsult characteristics associated with scheduling a nephrology appointment, including appointments scheduled initially and after provider communication. Multivariable logistic regression was adjusted for patient age, sex, and serum creatinine.

Characteristic	Scheduled for Nephrology Appointment		
	aOR	95% CI	*p*-Value
**Clinical Question Type***			
Management vs. diagnosis (ref)	2.07	1.25–3.45	0.005
Medication inquiry vs. diagnosis (ref)	0.14	0.03–0.64	0.01
**Patient Demographics**			
Age at referral	0.99	0.98–1.00	0.17
Female vs. male (ref)	0.77	0.51–1.17	0.22
**Kidney Function Severity**			
sCr	1.09	0.94–1.27	0.24

CI: confidence interval; OR: odds ratio; sCr: serum creatinine.
* 'Other' category (0.44%, *n* = 2/450) not included in multivariable analysis.
